# OCT-based diagnosis, management, and predictors of recurrent stent failure: a cohort study

**DOI:** 10.3389/fcvm.2025.1565676

**Published:** 2025-05-13

**Authors:** Giacomo Maria Cioffi, Pablo Lamelas, Mariam Shenouda, Jamie Halperin, Francesca Goffredo, Brian Patrick McGrath, Norman Said Vega Servin, Shamir R. Mehta, Sanjit S. Jolly, J. D. Schwalm, Madhu K. Natarajan, Nicholas Valettas, James L. Velianou, Michael B. Tsang, Natalia Pinilla-Echeverri, Matthew G. Sibbald, Tej N. Sheth

**Affiliations:** ^1^ Division of Cardiology, Hamilton General Hospital, Hamilton Health Sciences, McMaster University, Hamilton, ON, Canada; ^2^ Department of Cardiology, University and Hospital of Fribourg, Fribourg, Switzerland; ^3^ Health Research Methods, Evidence, and Impact, McMaster University, Hamilton, ON, Canada; ^4^ Department of Cardiology, Fundacion Favaloro, Buenos Aires, Argentina; ^5^ School of Medicine, McMaster University, Hamilton, ON, Canada; ^6^ Population Health Research Institute (PHRI), McMaster University, Hamilton, ON, Canada; ^7^ Department of Anesthesiology, University and Hospital of Fribourg, Fribourg, Switzerland

**Keywords:** optical coherence tomography, stent failure, in-stent restenosis, stent thrombosis, intracoronary imaging

## Abstract

**Background:**

Stent failure (SF) is a complication of percutaneous coronary intervention (PCI).

**Objectives:**

This study aimed to assess the relationship of the optical coherence tomography (OCT) determined cause of SF with time since stent implantation, treatment, and outcome.

**Methods:**

This retrospective study included patients who underwent an OCT evaluation for SF from January 2013 to July 2023. In-stent findings were evaluated on OCT including tissue proliferation, tissue type, underexpansion, thrombus, and multiple stent layers. The relationship between time to presentation, treatment, and outcome was assessed.

**Results:**

Of the 309 patients who underwent an OCT-guided PCI for SF, tissue proliferation was present in 228 (74%) and absent in 81 (26%). Among patients with tissue proliferation, OCT commonly showed lipidic neointima (*n* = 122, 54%), thrombus (*n* = 81, 36%), and underexpansion (*n* = 71, 31%). In patients without tissue proliferation, OCT commonly identified underexpansion (*n* = 58, 72%), thrombus (*n* = 55, 68%), and uncovered struts (*n* = 37, 46%). The mean time to SF was 6.89 ± 5.88 years with tissue proliferation and 2.98 ± 3.75 years without (*p* < 0.001). Patients with tissue proliferation were more likely to be treated with repeat stenting (78% vs. 60%, *p* < 0.001). Lipidic neointimal tissue and >1 layer of stent were predictors of target SF recurrence during a median 3 years of follow-up.

**Conclusion:**

In a large series of OCT-guided treatments of SF, tissue proliferation was more common, occurred later after stent implantation, and was more likely to be treated with repeat stenting than no-tissue proliferation. Lipidic neointimal tissue and >1 layer of stent were significant predictors of target SF during follow-up.

## Introduction

Despite technological advances in percutaneous coronary intervention (PCI) devices, pharmacological regimens, and PCI techniques, the risk of stent failure (SF) persists (1). SF may present as stable angina or as myocardial infarction (MI) and as either in-stent restenosis (ISR) or stent thrombosis (ST). Studies using optical coherence tomography (OCT) have shown the ability to identify a variety of mechanisms of SF including uncovered stent struts, malapposition, and tissue proliferation. By evaluating imaging characteristics, the nature of in-stent tissue can be further characterized as lipidic, fibrotic, or calcified. Imaging can also identify when stent underexpansion and thrombus are present. However, there are limited data on the impact of OCT findings on subsequent treatment and prognosis after the acute management of SF. Therefore, the objectives of this study were to characterize the cause of SF by OCT and to determine (1) the relationship between the cause of SF and the time since stent implantation, (2) the impact of the cause of SF on OCT on the PCI treatment approach, and (3) subsequent prognosis.

## Methods

This observational, retrospective study investigated patients with SF who underwent OCT-guided PCI at a Canadian academic center. The center performs approximately 3,000 PCI procedures annually, including roughly 100–200 cases of stent failure. This study was approved by the institutional ethics review board and conducted in compliance with the current version of the Declaration of Helsinki and ICH-GCP.

### Patient population

All consecutive patients treated for SF with OCT imaging between January 2013 and July 2023 were screened. Patients were eligible if they had at least one pretreatment OCT pullback. Patients were excluded if the OCT pullbacks were of insufficient quality to permit image analysis, if no definite cause of SF could be identified, or if clinical data were unavailable.

### Definition of stent failure

Stent failure was defined as a clinically driven angiographic finding demonstrating >50% reduction of the lumen diameter within a previously placed stent. Clinical indications for angiography included stable angina, unstable angina, NSTEMI, or STEMI.

### OCT: image analysis

OCT image analysis of the target stent failure (TSF) was performed using APTIVUE Software (Abbott Vascular Inc., Santa Clara, CA, USA). If images were not already correctly calibrated, they were recalibrated using the dedicated adjustment tool in the APTIVUE software. The images were analyzed by two investigators (GC and NV). In case of interpretation uncertainty, findings were confirmed by an additional senior reader. All cross-sectional images/areas [cross-sectional area (CSA)] were initially screened for quality assessment. Cases were deemed to be of insufficient image quality if any significant portion of the stent was outside of the image or if the image had poor quality caused by insufficient blood clearance, artifacts, or reverberation impeding assessment (2).

For each failed stent, lumen, stent area, and diameter measurements were made as follows: minimum lumen area (MLA) (mm^2^), stent area at MLA (mm^2^), neointimal area (mm^2^), stent length (mm), number of layers of stent, proximal and distal reference areas (mm^2^), and external elastic lamina (EEL) at proximal and distal reference segments (mm). Quantitative measurements were performed at 10 cross sections equally spaced throughout the stent length (1, 3, 4).

### OCT: cause and plaque assessment

The primary cause of stent failure was assessed at the MLA—or occlusion site—and within 5 mm proximally or distally of the MLA as related to the absence or presence of in-stent tissue proliferation. If the cause was unclear, or there was presence of a thrombus not allowing definitive cause identification, the cause was deemed indeterminate. In case of the absence of tissue, the cause of the stent failure was determined by the presence of one or more of the following findings: uncovered struts, malapposition, and/or underexpansion. Struts were defined as uncovered if tissue could not be identified above the stent struts. Malapposition was defined as the lack of contact between stent struts and vessel wall of at least ≥300 µm for a length of at least 3 mm in length. Underexpansion was defined as the stent area divided by the mean reference area of <80% if both references were available, <90% if only the distal reference was available, or <70% if only the proximal reference was available. The presence of a thrombus was also noted.

When tissue—defined as any tissue presence between the luminal contour and stent contour—was present, the OCT imaging features were used to further characterize the plaque as lipidic, fibrotic, calcified (sheet or nodular), or speckled/layered neointima. Neointima was defined as the tissue between the luminal contour and stent contour. Lipidic plaque was defined as signal-poor regions with diffuse borders and high attenuation (4). A fibrotic plaque was identified as a signal-rich region with low attenuation. A calcified plaque was defined as a well-delineated, signal-poor region with sharp borders. Speckled/layered neointima was defined as a heterogeneous, layered pattern with both signal-rich and signal-poor regions and low attenuation. In addition, the presence of a thrombus and/or underexpansion was also assessed.

Plaque morphology was also assessed quantitatively at 10 evenly spaced cross sections throughout the stent. On these cross sections, the proportion of diseased quadrants and the predominant plaque type in each quadrant were recorded. The percentage of each plaque type among diseased quadrants was determined. For further and additional OCT definitions and acronyms, please refer to the Supplementary Material.

### OCT-based treatment

The local institutional OCT-based treatment protocol for stent failure was to use a balloon-only strategy intervention in patients without tissue proliferation when feasible. Drug-eluting stent (DES) implantation or the use of a drug-coated balloon (DCB) was preferred for patients presenting with evidence of tissue proliferation. However, the final treatment strategy of the failed stent was at each operator's discretion considering both OCT and clinical factors.

### Baseline characteristics and clinical and angiographical outcomes

Baseline and angiographical characteristics and clinical outcomes occurring during follow-up were collected from clinical records for the longest available follow-up (MS and JH). The primary outcome was target stent failure, defined as a clinically driven angiographic finding with the presence of significant restenosis or thrombosis of the previously implanted stent. Secondary outcomes included myocardial infarction (MI), recurrent stent failure, repeat revascularization with PCI or coronary artery bypass grafting (CABG), all-cause death, cardiovascular death, and major cardiovascular events (MACE), defined as a composite of the individual secondary outcomes. Procedural safety was assessed by collecting periprocedural complications including coronary artery dissections and perforations (based on Ellis grade), no reflow, acute vessel closure, acute kidney injury, or bleeding events (based on BARC criteria).

### Statistical analysis

The categorical variables were summarized using frequencies and proportions (percentage) and were compared using the chi-square statistics or Fisher's exact test, as appropriate. Normally distributed continuous data were summarized as mean and standard deviation and compared using the Student's *t*-test. Non-normally distributed continuous variables were reported as median with interquartile range (IQR, 25th and 75th percentiles) and were compared using Mann–Whitney *U*-test or non-parametric Wilcoxon rank-sum test. The Kaplan–Meier method was used to estimate the cumulative probability of freedom from target stent failure. A univariable and multivariable Cox proportional hazards regression model was used to evaluate and identify OCT predictors of recurrence of stent failure. The results of the model are presented as the hazard ratio (HR) and 95% CI. The Prentice Williams and Peterson gap time (PWP-GT) model was used. Using the estimates from the multivariable model, we estimated the population attributable fraction (PAF) which represents the proportion of recurrent stent failures that would be prevented if the risk factor was eliminated. All tests were two-sided with a 5% significance level. All statistical analyses were performed using Stata Software (StataCorp. 2023. *Stata Statistical Software: Release 18*. College Station, TX: StataCorp LLC) and SPSS software, Version 22.0 (IBM Corp., Armonk, NY, USA).

## Results

**Figure 1:**
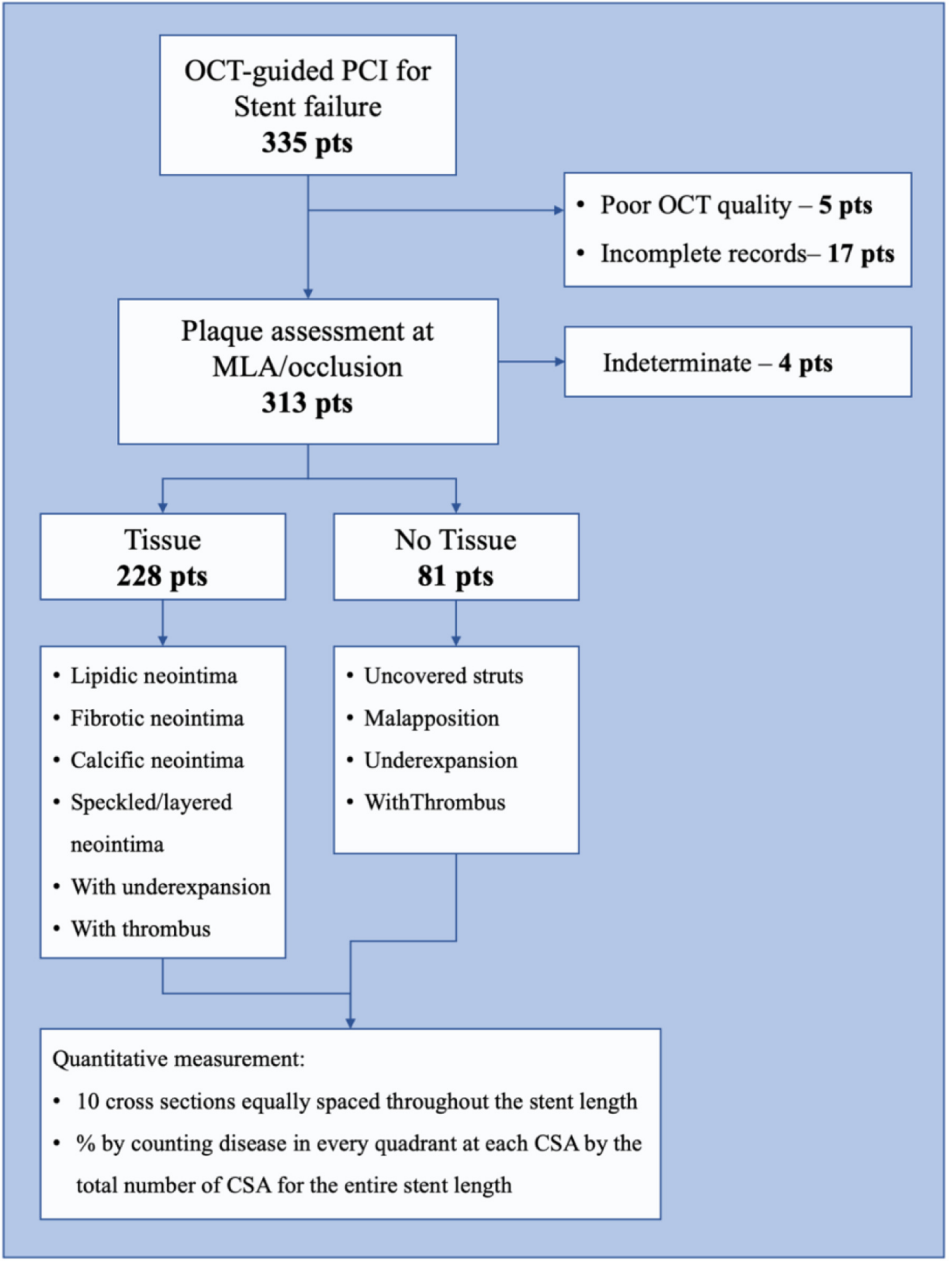
Study workflow. This figure shows the study workflow of the current study. Of all the screened patients presenting OCT-guided PCI for stent failure, 335 patients were identified. After exclusion criteria were applied (poor OCT quality, 5 patients; incomplete data records, 17 patients), 313 patients underwent analysis, of whom 4 patients were excluded because of indeterminate stent failure cause. The 309 remaining patients underwent a qualitative OCT assessment of stent failure caused by identifying “tissue” or “no tissue” causes and characterizing them accordingly. Furthermore, each patient underwent a quantitative assessment.

**Figure 2:**
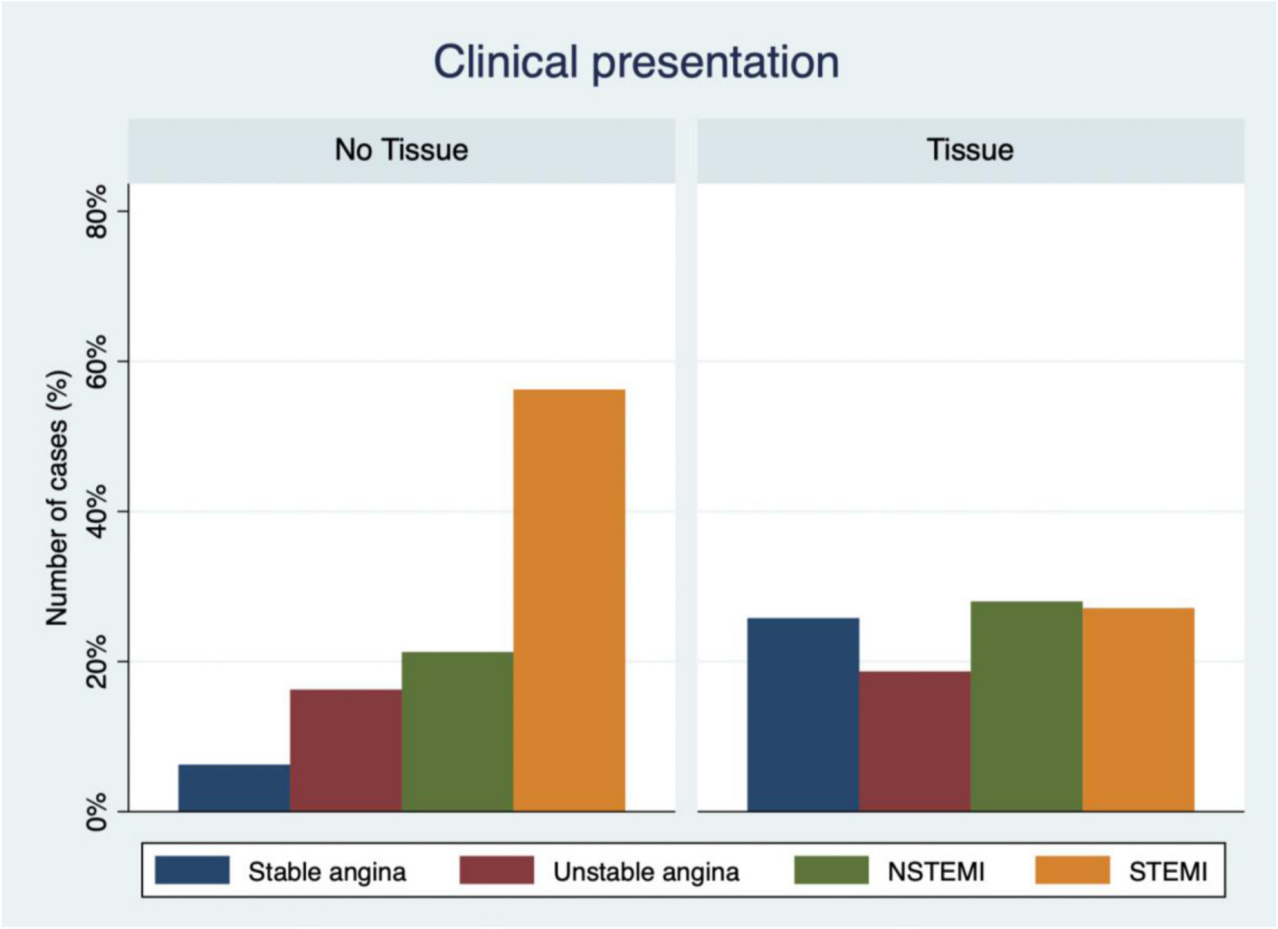
Clinical presentation of stent failure and OCT identification of in-stent tissue presence or absence. This bar chart graphic represents the clinical presentation distributions among the two groups of stent failure. Each column represents the frequency of presentation of stable angina, unstable angina, NSTEMI, and STEMI for the in-stent tissue absence or presence.

Of the 335 patients with SF treatment guided by OCT during the study period, 5 were excluded because of non-acceptable OCT image quality, 17 were excluded due to lack of follow-up, and 4 were then excluded by the analysis because of no clear cause of stent failure (Figure 1). Of the 309 patients in the analysis, tissue proliferation was found in 228 (74%) and no-tissue proliferation in 81 (26%). Patients with tissue proliferation were more likely to present with stable angina and dyslipidemia and less likely to present with STEMI (Figure 2). Time to presentation after index PCI was also longer at 6.89 ± 5.88 years vs. 2.98 ± 3.75 years (*p* < 0.001). Further group characterization is presented in Table 1.

**Table 1:** Baseline characteristics.

Baseline characteristics	Overall (*N* = 309)	No tissue (*N* = 81)	Tissue (*N* = 228)	*p*-value
Age at time of stent failure, years ± SD	66 ± 11	67 ± 12	66 ± 11	0.326
Time from index PCI to stent failure, years (IQR)	5.88 ± 5.67	2.98 ± 3.75	6.89 ± 5.88	<0.001
Female, *n* (%)	60 (20)	14 (18)	46 (20)	0.592
BMI, kg/m^2^ (±SD)	28.7 ± 6.1	28.5 ± 4.7	28.8 ± 6.5	0.714
Cardiovascular risk factors, *n* (%)				
Arterial hypertension	278 (90)	69 (85)	209 (92)	0.095
Diabetes mellitus	123 (40)	33 (41)	90 (40)	0.885
Dyslipidemia	284 (92)	70 (86)	214 (94)	<0.05
Current or past smoking	202 (66)	46 (57)	156 (69)	0.052
Family history of CAD	133 (44)	32 (41)	101 (45)	0.462
Comorbidities, *n* (%)				
Previous MI, *n* (%)	262 (87)	68 (86)	194 (86)	0.906
Previous CABG, *n* (%)	40 (13)	9 (11)	31 (14)	0.589
History of stroke, *n* (%)	24 (8)	5 (6)	19 (9)	0.455
History of PVD, *n* (%)	37 (12)	8 (10)	29 (13)	0.429
History of COPD, *n* (%)	43 (14)	10 (13)	33 (15)	0.630
Clinical presentation at time of stent failure, *n* (%)				
Stable angina	63 (21)	5 (6)	58 (26)	<0.001
Unstable angina	55 (18)	13 (16)	42 (19)	0.629
NSTEMI	80 (26)	17 (21)	63 (28)	0.238
STEMI	106 (35)	45 (56)	61 (27)	<0.001
LVEF at time of stent failure, % (±SD)	46 ± 11	41 ± 12	47 ± 10	<0.001
Ischemia testing at time of stent failure, *n* (%)				
Treadmill stress test, *n* (%)	28 (9)	4 (5)	24 (11)	0.136
MIBI, *n* (%)	24 (8)	6 (7)	18 (8)	0.692
Stress echocardiography, *n* (%)	24 (8)	3 (4)	23 (10)	0.192
Laboratory tests at time of stent failure				
eGFR, ml/min/1.73 m^2^ (±SD)	78 ± 22	76 ± 24	79 ± 21	0.323
Hemoglobin, g/l (±SD)	137 ± 18	134 ± 21	137 ± 16	0.183
Antiplatelets at time of stent failure, *n* (%)				
None	9 (3)	1 (1)	8 (4)	0.295
Aspirin only	45 (15)	10 (13)	35 (16)	0.508
Clopidogrel only	5 (2)	1 (1)	4 (2)	0.750
DAPT (with clopidogrel)	129 (42)	32 (40)	97 (43)	0.629
DAPT (with ticagrelor)	119 (39)	36 (45)	83 (37)	0.201
Statin therapy, *n* (%)	268 (88)	70 (86)	198 (88)	0.641
Ezetimibe therapy, *n* (%)	56 (18)	10 (13)	46 (21)	0.111
PCSK-9 therapy, *n* (%)	1 (0.5)	–	1 (0.5)	1.000

Data are mean (SD, standard deviation), median (IQR, interquartile range), or number (*n*, number; %, percentage), as appropriate. PCI, percutaneous coronary intervention; ISR, in-stent restenosis; BMI, body mass index; CAD, coronary artery disease; LVEF, left ventricular ejection fraction; MI, myocardial infarction; CABG, coronary artery bypass grafting; PVD, peripheral vascular disease; COPD, chronic obstructive pulmonary disease; NSTEMI, non-ST-elevation myocardial infarction; STEMI, ST-elevation myocardial infarction; MIBI, myocardial perfusion sestamibi imaging; eGFR, estimated glomerular filtration rate; DAPT, dual-antiplatelet therapy.

**Figure 3:**
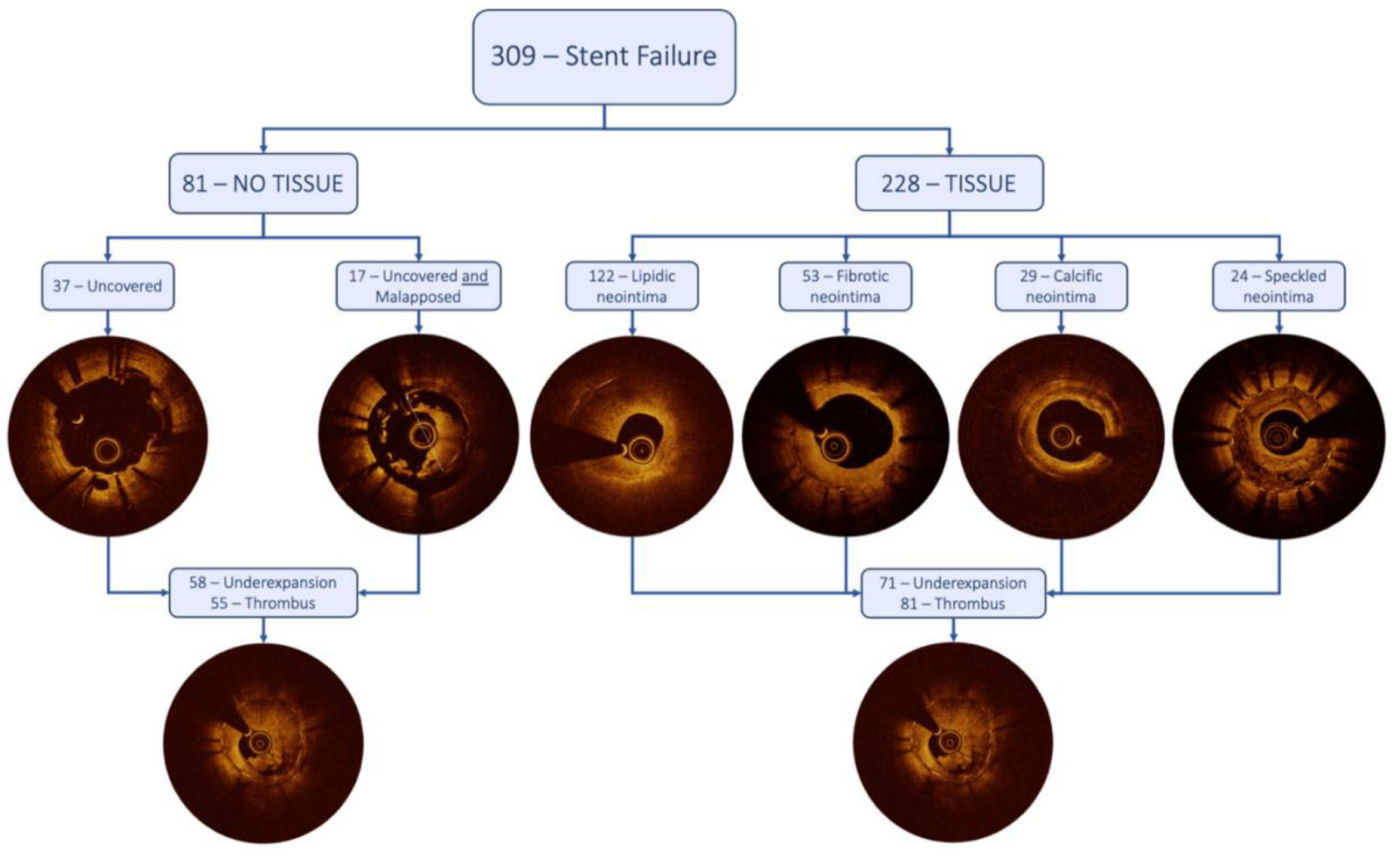
Central illustration. This figure visually shows the differentiation of the two different stent failure groups and their subgroups by qualitative analysis with their respective frequencies.

On OCT analysis (Table 2), qualitative assessment demonstrated that the most frequent plaque composition among the tissue group was the lipidic neointima in 122 (54%) cases, followed by the fibrotic neointima in 53 (23%), calcific neointima in 29 (13%), and speckled/layered neointima in 24 (11%). Underexpansion or thrombus was an additional finding in 71 (31%) and 81 (36%) cases, respectively (Figure 3). Quantitative assessment performed throughout the stent paralleled the qualitative MLA site assessment (Table 3). For example, in patients with fibrotic neointima at MLA, most of the in-stent plaque was fibrotic, and in patients with speckled neointima at MLA, the most common in-stent plaque type was also speckled.

**Table 2:** OCT plaque characterization.

OCT plaque characterization	Overall (*N* = 309)
Cause of stent failure, *n* (%)	
No tissue	81 (26)
Uncovered struts	37 (46)
Uncovered struts and malapposition	17 (21)
Underexpansion	58 (72)
With thrombus	55 (68)
Tissue	228 (74)
Lipidic neointima	122 (54)
Fibrotic neointima	53 (23)
Calcific neointima	29 (13)
Speckled/layered neointima	24 (11)
With underexpansion	71 (31)
With thrombus	81 (36)

Data are mean (SD, standard deviation), median (IQR, interquartile range), or number (*n* = number; % = percentage), as appropriate. OCT, optical coherence tomography.

**Table 3:** Quantitative plaque assessment over the tissue group of stent failure.

	Lipidic (*N* = 122)	Fibrotic (*N* = 57)	Calcific (*N* = 29)	Speckled (*N* = 24)
Maximum arc at MLA, degrees (±SD)	294 ± 81	333 ± 70	240 ± 94	334 ± 62
Calcified nodule, *n* (%)			6 (20)	
Maximum calcium thickness, mm (±SD)			0.93 ± 0.31	
Quantitative assessment, % (±SD)				
Lipid	36 ± 21	7 ± 10	4 ± 8	6 ± 7
Fibrotic	31 ± 20	55 ± 20	39 ± 23	30 ± 18
Calcific	4 ± 7	4 ± 9	38 ± 17	2 ± 6
Speckled	2 ± 8	1 ± 3	–	32 ± 18

Data are mean (SD, standard deviation), median (IQR, interquartile range), or number (*n*, number; %, percentage), as appropriate. MLA, minimal lumen area.

In the no-tissue group, underexpansion and uncovered struts were the most represented findings with 58 (72%) and 37 (46%) cases, respectively. Uncovered struts combined with significant malapposition were present in 17 (21%) cases, and thrombus was shown in 58 cases (68%) (Table 2). Stents without tissue proliferation had a larger lumen area and smaller stent area at the MLA site compared with stents with tissue proliferation. Multiple stent layers were seen in 16% and 18% of both groups. Additional OCT measurements are shown in Table 4.

**Table 4:** Pre-PCI OCT measurements.

OCT measurement	Overall (*N* = 309)	No tissue (*N* = 81)	Tissue (*N* = 228)	*p*-value
MLA at time of OCT, mm^2^ [IQR]	1.87 [1.31]	2.35 [1.83]	1.81 [1.12]	<0.001
Stent area at MLA, mm^2^ (±SD)	6.86 ± 2.44	6.23 ± 2.79	7.09 ± 2.27	<0.05
Tissue proliferation area, mm^2^ (±SD)	–	–	5.09 ± 2.10	–
Proximal reference area, mm^2^ [IQR]	7.74 [4.38]	7.78 [4.48]	7.66 [4.33]	0.945
EEL at proximal reference area, mm^2^ [IQR]	12.07 [6.17]	12.03 [6.06]	12.07 [6.41]	0.493
Distal reference area, mm^2^ [IQR]	4.89 [2.81]	4.93 [2.63]	4.88 [2.94]	0.901
EEL at distal reference area, mm^2^ [IQR]	7.65 [3.93]	7.52 [3.65]	7.73 [3.91]	0.607
Stent length, mm (±SD)	31 ± 15	32 ± 14	31 ± 15	0.569
>1 stent layer, *n* (%)	55 (18)	13 (16)	42 (18)	0.501

Data are mean (SD, standard deviation), median (IQR, interquartile range), or number (*n*, number; %, percentage), as appropriate. MSA, minimal stent area; CSA, cross-sectional area. ^‡^Stent eccentricity index = minimum diameter / maximum diameter.

**Figure 4:**
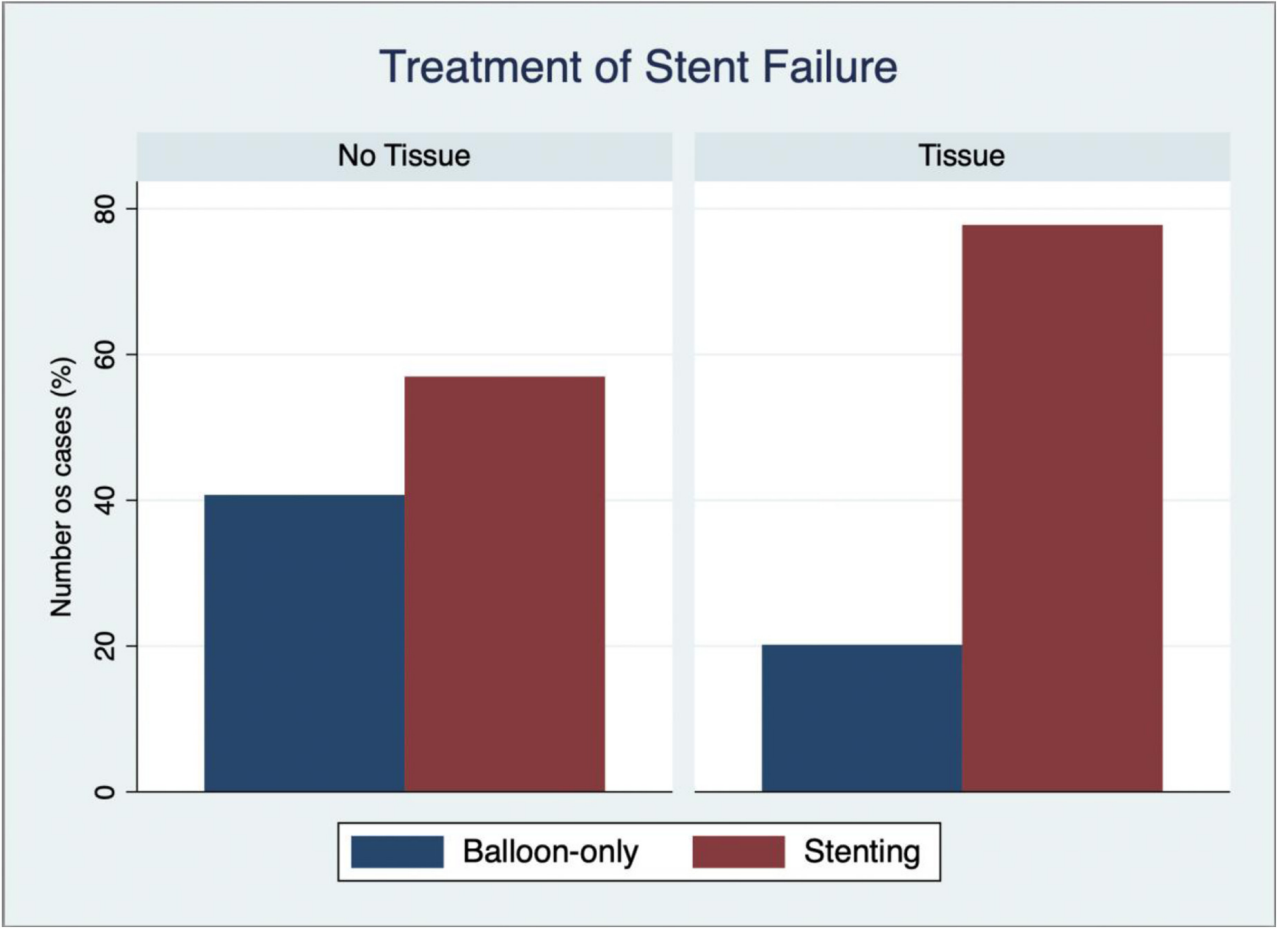
Treatment of stent failure based on the presence or absence of in-stent tissue as identified by OCT. This bar chart graphic shows the two different treatment strategies of balloon-base or stent-based only approach between the different stent failure groups—tissue absence and presence, respectively.

At angiography, there was no difference in lesion location. TIMI 3 flow was more common in the tissue group, and thrombus aspiration was less likely to be used (Table 5). Balloon-based only treatment [semi-compliant (SC), non-compliant (NC), cutting/scoring, and/or DCB] was more commonly used in the no-tissue group compared with the tissue group (43% vs. 20%, *p* < 0.001), whereas stenting was higher in the tissue group compared with the no-tissue group (78% vs. 60%, *p* < 0.001) (Figure 4). The periprocedural rate of complications was very low with only three (1%) patients suffering no reflow and one presenting abrupt vessel closure. Three patients suffered from acute kidney failure following PCI and three presented a BARC 3a bleeding complication (access site). There were no dissections or perforations.

**Table 5:** Angiographic characteristics and treatment.

Angiographic characteristics at time of stent failure	Overall (*N* = 309)	No tissue (*N* = 81)	Tissue (*N* = 228)	*p*-value
Culprit vessel, *n* (%)				
Left main, *n* (%)	3 (1)	0 (0)	3 (1)	0.297
Left anterior descending, *n* (%)	170 (55)	49 (60)	121 (53)	0.280
Left circumflex, *n* (%)	48 (16)	12 (15)	36 (16)	0.813
Right coronary artery, *n* (%)	74 (24)	15 (19)	59 (26)	0.171
Vein graft, *n* (%)	16 (5)	4 (5)	12 (5)	0.891
Initial TIMI flow, *n* (%)				
0	77 (30)	31 (43)	46 (24)	<0.05
1	16 (6)	7 (10)	9 (5)	0.132
2	49 (19)	12 (17)	37 (20)	0.603
3	120 (46)	22 (31)	98 (52)	<0.05
Treatment before OCT, *n* (%)				
None	95 (31)	26 (33)	69 (31)	0.711
Balloon dilatation	201 (66)	52 (66)	149 (66)	0.949
Rotablation	7 (2)	3 (4)	4 (2)	0.303
Thrombus aspiration	16 (5)	10 (13)	6 (3)	0.001
Treatment of stent failure, *n* (%)				
Balloon-based only treatment	79 (26)	35 (43)	46 (20)	<0.001
SC or NC only	37 (12)	24 (30)	15 (7)	
DCB	40 (13)	11 (14)	29 (13)	
Stenting	220 (72)	45 (60)	179 (78)	<0.001
Direct stenting	15 (5)	-	15 (7)	
After lesion preparation	182 (59)	42 (52)	144 (63)	
Hybrid (DES and DCB)	30 (10)	3 (4)	27 (12)	
Postdilatation	174 (57)	36 (45)	138 (61)	<0.05
CABG	4 (1)	1 (1)	3 (1)	1.000
Final TIMI flow, *n* (%)				
2	3 (1)	1 (1)	2 (1)	1.000
3	294 (99)	78 (99)	216 (99)	1.000

Data are mean (SD, standard deviation), median (IQR, interquartile range), or number (*n*, number; %, percentage), as appropriate. ISR, in-stent restenosis; TIMI, thrombolysis in myocardial infarction; OCT, optical coherence tomography; SC, semi-compliant; NC, non-compliant; DCB, drug-coated balloon; DES, drug-eluting stent; CABG, coronary artery bypass grafting.

### Clinical outcomes

After a mean follow-up of 36 months, a total of 29 (10%) patients presented with the primary outcome of recurrent stent failure (Table 6). Of these, 24 (8%) patients were revascularized via PCI and 14 (5%) underwent CABG. There was a statistically significant difference between the no-tissue group and tissue group among the recurrence of stent failure with 3 (4%) and 26 (12%) cases, respectively (*p* < 0.05). A total of 76 (25%) patients experienced the composite secondary outcome of MACE. Of these, 50 (16%) presented a new MI, and 15 (5%) patients died, of whom 5 (2%) had a cardiovascular death.

**Table 6:** Clinical outcomes.

Clinical outcomes	Overall (*N* = 309)	No tissue (*N* = 81)	Tissue (*N* = 228)	Non-lipidic (*N* = 106)	Lipidic (*N* = 122)	*p*-value (no tissue vs. tissue)
Time to follow-up, months (±SD)	36 ± 32	34 ± 32	36 ± 32	36 ± 30	36 ± 33	0.547
Target stent failure, *n* (%)	29 (10)	3 (4)	26 (12)	8 (8)	18 (15)	<0.05
Target stent revascularization, *n* (%)						
PCI, *n* (%)	24 (8)	2 (3)	22 (10)	8 (8)	14 (12)	<0.05
CABG, *n* (%)	14 (5)	1 (1)	13 (6)	6 (6)	7 (6)	0.124
MACE, *n* (%)	76 (25)	20 (25)	56 (25)	26 (25)	30 (25)	0.981
New MI, *n* (%)	50 (16)	12 (15)	38 (17)	15 (15)	23 (19)	0.684
All-cause death, *n* (%)	15 (5)	7 (9)	8 (4)	3 (3)	5 (4)	0.076
Cardiovascular death, *n* (%)	5 (2)	2 (3)	3 (1)	3 (3)	0 (0)	0.610

Data are mean (SD, standard deviation), median (IQR, interquartile range), or number (*n*, number; %, percentage), as appropriate. MACE, major adverse cardiovascular events, defined as a composite of myocardial infarction, recurrent stent failure and repeat revascularization with PCI or CABG; MI, myocardial infarction; PCI, percutaneous coronary intervention; TSF, target stent failure, defined as clinically driven angiogram with the presence of significant restenosis or thrombosis of the previously implanted stent; CABG, coronary artery bypass grafting.

### Predictors of stent failure

**Figure 5:**
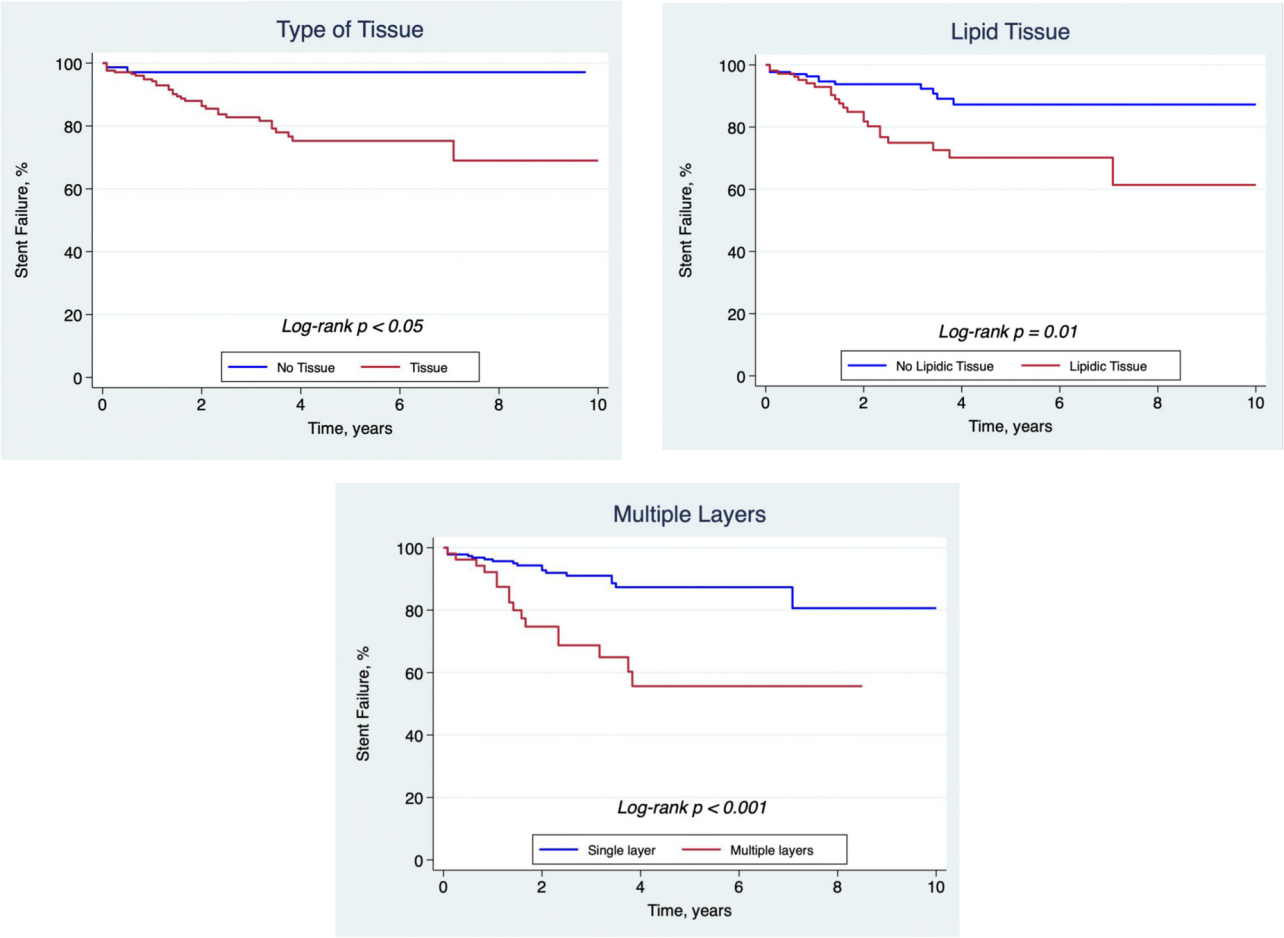
Kaplan–Meier curves. This figure shows the freedom from stent failure within the presence or absence of tissue proliferation, the presence of lipidic tissue substrate, and multiple layers of stent (>1) over time.

In a univariable analysis, the presence of lipidic neointima, stent underexpansion with tissue, and >1 layer of stent were significantly associated with stent failure recurrence at follow-up (Table 7 and Figure 5). Multivariable analysis demonstrated that lipidic neointima (HR 2.785, 95% CI: 1.304–5.948; *p* = 0.008) and >1 stent layer (HR 3.441, 95% CI: 1.566–7.562; *p* = 0.002) were independent predictors for recurrent stent failure. The population attributable fraction (PAF) for lipid neointima and multiple layers was 41% and 30.5%, respectively.

**Table 7:** Cox regression—univariable and multivariable model.

Univariable	HR	Lower	Upper	*p*-value
Lipidic tissue	2.590	1.223	5.486	<0.05
Fibrotic tissue	0.947	0.361	2.482	0.912
Calcific tissue	0.339	0.046	2.498	0.289
Speckled tissue	0.899	0.214	3.782	0.885
Underexpansion	1.379	0.665	2.858	0.388
Underexpansion without tissue	0.510	0.154	1.685	0.269
Underexpansion with tissue	2.092	0.998	4.385	0.051
>1 stent layer	3.432	1.650	7.138	<0.05
Uncovered struts	0.041	0.000	5.060	0.193
Malapposition	0.044	0.000	19.520	0.315
Multivariable				
Lipidic tissue	2.785	1.304	5.948	<0.05
>1 stent layer	3.441	1.566	7.562	<0.05
Underexpansion with tissue	1.309	0.590	2.905	0.508

## Discussion

This study reports one of the largest series of patients presenting with stent failure evaluated with OCT. The major findings were as follows: (1) tissue proliferation was the most common cause of stent failure with lipidic neointima representing more than half of cases, (2) time to stent failure from the index procedure was significantly longer in patients with tissue proliferation, (3) OCT findings impacted the treatment strategy with more repeated stenting and post-dilation in stents with tissue proliferation compared with without, and (4) the presence of lipidic neointima and >1 layer of stent were significant independent predictors of recurrent stent failure.

### Tissue proliferation and time to presentation

The evolution of DES has significantly reduced acute thrombotic events and restenosis rates. However, late stent failure persists, as several trials showed a growing incidence of target lesion revascularization over time across all DES generations (5). The mean time to stent failure in our series reflects presentations typically beyond the first year after implantation, i.e., a mean of almost 2.98 years for patients without tissue proliferation and 6.89 years for patients with tissue proliferation. This may be explained by (a) changes in presentation time for stent failure with failure delayed by contemporary stent technologies and/or (b) operator choice to use OCT preferentially for stents presenting with very late failure (>12 months from implantation). Notably, tissue proliferation etiologies presented later than no-tissue patients, likely reflecting the beneficial effect of DES to avoid early stent tissue proliferation. The preponderance of neoatherosclerosis vs. fibrotic neointima is also typical of this time point as demonstrated in two OCT-based studies that observed elevated frequencies of neoatherosclerosis with restenosis beyond 3 years (6, 7).

### Treatment strategy

Imaging-based management of stent failure has been proposed in the literature based on the predominant mechanism of failure (8–16). However, the impact of imaging findings on treatment choice has not been previously demonstrated. In this study, we observed significantly higher use of a balloon-based only strategy in patients presenting no-tissue proliferation as a cause of stent failure.

Conversely, when tissue proliferation was present, we observed significantly higher DES use compared with a ballon-based only strategy, suggesting that operators were using imaging findings to select therapy. This trend was also observable in the group presenting lipidic neointima as the main attributable cause of stent failure.

Several studies and meta-analyses consistently reported DES as the most effective treatment for stent failure due to tissue proliferation (17–20). Recently, this has also been recommended as a Class IA indication by the 2024 European Society of Cardiology guidelines for the management of chronic coronary syndromes (21).

Although this confirms the alignment with such studies by the operators of the present study, it contrasts with our results that showed that the presence of one or more stent layers was a predictor of stent failure recurrence.

The choice of implanting an additional layer of stent might have been dictated, additionally and other than by operator's preference, by patients' clinical presentation—especially STEMI and NSTEMI—suggesting that the concomitant presence of a thrombus material had its weight on the procedural choice.

Notably, we did not observe any specific trend or change in the treatment strategy of patients presenting with tissue proliferation—and especially lipid neointima—over the study period.

Although new evidence and studies are supporting the use of the drug-coated balloon technology and the limitation of additional layer implantation, further studies are needed to establish their role in the treatment of ACS-related stent failure due to plaque ruptures and erosions.

### Predictors of recurrent events

Patients presenting with stent failure have an increased risk of recurrent stent failure events. Several studies have assessed the drivers of these recurrent events in different contexts. Recurrent stent failures have been identified to be significantly related to underexpansion and associated with worst outcomes in patients presenting three layers of stent (15, 22, 23). Lipidic neointima was also shown to be an independent predictor of target lesion revascularization in a recent OCT study (24). In our analysis, a Cox univariable and multivariable logistic regression model demonstrated that the presence of lipidic neointima and >1 layer of stent were significant independent predictors of recurrence of stent failure. Furthermore, using the population attributable fraction (PAF) approach, we observed that two-thirds of future stent failures could be explained by the presence of lipid tissue or >1 stent layer in the indexed stent failure assessment. Among patients with multiple layers of stent, further understanding of lesions or patient characteristics that are best served with balloon-based strategies rather than additional layers of a stent is needed. Among patients with lipidic neointima, aggressively managing risk factors associated with the progression of disease, especially dyslipidemia should be a key part of the management strategy. It is notable that in this series, potentially correctable factors, such as underexpansion and the type of treatment strategy, were not predictors of recurrent events and that population attributable risk of recurrence was largely related to variables not correctable by OCT (multiple stent layers and lipidic neointima).

### Limitations

Several, important limitations are worth mentioning. First, this was a retrospective, single-center, and observational study. Second, although very important, no data on the total number of stent failure treated within the study time were able to be retrieved. The lack of a dedicated PCI registry of stent failure did not enable us to retrieve such worthy information. Accordingly, it was impossible to retrieve information on the stent platform used at the index procedure (bare metal stent, generation of DES, etc.). Third, selection bias might have been particularly relevant, as the decision to perform OCT in the event of stent failure was entirely at the operator's discretion. Fourth, treatment before the very first OCT pullback, especially those with balloon dilatation and adjunctive tools (i.e., rotational atherectomy), may have influenced the initial OCT assessment by modifying the first assessable MLA. Fifth, although analyzed by two operators and intravascular imaging experts not involved in the studied procedures, the absence of an independent imaging core laboratory for OCT analysis may raise concerns about potential bias in the interpretation of imaging findings. Sixth, the lack of an independent clinical event adjudication committee may have introduced bias in the classification of clinical outcomes.

## Conclusions

In a large series of OCT-guided treatments of stent failure, tissue proliferation was more common than no-tissue proliferation, occurred later after index stent implantation, and was more likely to be treated with stenting. The presence of >1 stent layer and lipidic tissue proliferation independently predicted the recurrence of events during follow-up.

## Data Availability

The raw data supporting the conclusions of this article will be made available by the authors, without undue reservation.
