# Supplementary material for: OCT-based diagnosis, management, and predictors of recurrent stent failure: a cohort study
Source: Front Cardiovasc Med. 2025 May 13;12:1565676. doi: 10.3389/fcvm.2025.1565676 (PMC12106419; doi:10.3389/fcvm.2025.1565676)
Supplement: Supplementary file 1 [file Supplementaryfile1.pdf]

Supplementary Material

OCT Definitions

Neointima is defined as the tissue between the luminal contour and stent contour.

Fibrotic neointima is identified as signal-rich regions with low attenuation.

Calcified neointima is defined as a well-delineated, signal-poor region with sharp borders.

Lipid neointima is defined as signal-poor regions with diffuse borders and high attenuation.(4)

Speckled or Layered neo-intima: heterogeneous, layered pattern with both signal-rich and -poor regions and low-attenuation.

Neoatherosclerosis is defined as the lipid or calcified neointima.(1)

Calcium results in low backscattering, low attenuation, and high penetration of the light source with the appearance of a signal poor (dark) region on OCT imaging. In addition, as calcium has a low-backscattering property compared to that of fibrous tissue (highly backscattering) the OCT signal drops suddenly at the borders between fibrous and calcified tissue resulting in sharply defined borders of the calcium deposits.

Calcium deposits might be of different types:

- **Superficial**: if the luminal leading edge of calcification is located within 0.5 mm from the lumen.
- **Deep**: if the luminal leading edge of calcification is >0.5 mm from the lumen. In some cases, the inner border of calcification can be identified but the outer border cannot.
- **Nodular**: protruding nodular calcification with dorsal shadowing

Calcium fracture (CF) is defined as a new disruption or discontinuity in the calcium sheet. (2) CF can be further characterized depending how wide and deep it is.

Calcium depth is the maximum thickness measured from the inner calcium layer to outermost visible layer.

Calcium length is the total number of calcium-containing slices multiplied by the frame interval.

Plaque: Loss of layered appearance, narrowing of lumen.

Ruptured plaque is defined as an intimal interruption and cavity formation in the plaque.

Red thrombus is a lumen-protruding mass with irregular borders, high-backscattering and low-penetration properties. (3, 4)

White thrombus is a lumen-protruding mass with irregular borders, low-backscattering and high-penetration properties.

Fibrous cap: Signal-rich band overlying signal-poor region.

Thin-cap fibroatheroma (TCFA) is considered when the fibrous cap thickness is ≤65 μm in the lipid-rich plaque.

Macrophages infiltration is defined as “bright spots” with very high signal variance from the surrounding tissue.

Vasa vasorum are microchannels with tiny black holes of 50-100 μm dimensions.(5)

Intima layer: Signal-rich layer nearest lumen.

Internal elastic lamina (IEL): Signal-rich band (~20 μm) between the intima and media.

Media layer: Signal-poor middle layer.

External elastic lamina (EEL): Signal-rich band (~20 μm) between the media and adventitia.

Adventitia layer: Signal-rich, heterogeneous outer layer.

Maximum and minimum lumen diameters: the longest and shortest lumen distances measured through the center of the lumen.

Minimum lumen area (MLA): smallest cross-sectional area (CSA) in the lesion segment.

Reference lumen area: is calculated by tracing the luminal contour on the proximal and distal reference segments (the largest area within 5 mm from the edge of lesion).

Lumen area stenosis: (the reference lumen CSA − minimal lumen CSA)/reference lumen CSA ×100.

Stent expansion (SE): minimum stent CSA in correlation with reference lumen CSA (proximal, distal, or average).

Stent underexpansion is defined when the MSA/mean (proximal and distal) reference area is <80% if both references were available, <90% if only the distal reference is available, or <70% if only the proximal reference is available.

Stent CSA: area circumscribed by the stent border.

Minimum stent CSA = smallest stent CSA.

Maximum and minimum stent diameters: the longest and shortest diameters measured through the center of the stent.

In-stent lumen area stenosis = (the reference lumen CSA − minimal lumen in-stent CSA)/reference lumen CSA ×100.

Neointimal area = Stent CSA − lumen CSA.

Acute lumen gain is defined as minimal lumen diameter (MLD) post balloon angioplasty minus baseline MLD in millimeters (mm) or minimum lumen area (MLA) post PCI minus baseline MLA in mm^2.

Stent Eccentricity index (SEI) is calculated as the ratio of minimum and maximum stent diameter per cross section.

1. Lee SY, Shin DH, Mintz GS, Kim JS, Kim BK, Ko YG, et al. Optical coherence tomography-based evaluation of in-stent neoatherosclerosis in lesions with more than 50% neointimal cross-sectional area stenosis. EuroIntervention. 2013;9(8):945-51.
2. Kubo T, Shimamura K, Ino Y, Yamaguchi T, Matsuo Y, Shiono Y, et al. Superficial Calcium Fracture After PCI as Assessed by OCT. JACC Cardiovasc Imaging. 2015;8(10):1228-9.
3. Kume T, Akasaka T, Kawamoto T, Ogasawara Y, Watanabe N, Toyota E, et al. Assessment of coronary arterial thrombus by optical coherence tomography. Am J Cardiol. 2006;97(12):1713-7.
4. Jang IK, Tearney GJ, MacNeill B, Takano M, Moselewski F, Iftima N, et al. In vivo characterization of coronary atherosclerotic plaque by use of optical coherence tomography. Circulation. 2005;111(12):1551-5.
5. Takano M, Yamamoto M, Inami S, Murakami D, Ohba T, Seino Y, et al. Appearance of lipid-laden intima and neovascularization after implantation of bare-metal stents extended late-phase observation by intracoronary optical coherence tomography. J Am Coll Cardiol. 2009;55(1):26-32
